# Appendix to the Case of Malformation in a Fœtus
*The words "Unusual Conformation," in the title of the plate, should have been "Extraordinary Malformation," agreeably to that of the case. In page 397, line 9 from the bottom, for "on the right side," read "on the left side."


**Published:** 1800-06

**Authors:** T. Pole


### *Appendix to the Case of Malformation* in a Fœtus.*

### By T. POLE. (See Page 397.)

IN consequence of this phænomenon being thrown before the public, through the medium of the Medical and Physical Journal, it has, agreeably to my wishes, met the eye of my ingenious friend, Charles Cooke, surgeon and practitioner in midwifery, in the city of Gloucester, (to whom I am indebted for this and another extraordinary lusus naturæ) who has obliged me with further particulars in the following letter:

"DEAR SIR,

"HAVING just read (in the Medical and Physical Journal) your Case of Malformation in a Fœtus, I am no longer surprised at your silence.

"Soon after that fœtus was forwarded, I requested a medical friend in London to call and inform you of it; and, if you wished to know the particulars, I would readily answer your queries.

"As I had not heard from you, I thought the parcel never came to hand; however, I am glad to find you did receive it, and will give a brief account of the presentation, labour, &c.

"In January last I was called to Mrs. ——; she had regular strong labour pains; I found the os uteri fully dilated, and the membranes distended; I could feel the action of the extremities of the fœtus within them, and labour appeared to be in that forward state, which required nothing but rupturing the membranes to produce a quick delivery, provided it should prove a footling case.

"When I ruptured the membranes, both feet immediately followed the discharge of the waters, and the other parts of the child progressively succeeded by a continuance (with hardly any intermission) of strong labour pains, so that I had no time to regard the manner in which the child was protruding, for, before I could have supposed it possible, the head was also expelled. The latter circumstance, added to this being a first child, greatly surprised me; and upon passing my hand higher, to ascertain the cause of such an easy expulsion of the head, I thought, from what I then felt, that the violent efforts of the mother

* The words "Unusual Conformation," in the title of the plate, should have been "Extraordinary Malformation," agreeably to that of the case.

In page 397, line 9 from the bottom, for "on the right side," read "on the left side."

mother had protruded the head entangled in the uterus. I was soon relieved from my apprehensions by feeling the ball upon the left side, and the funis on the same side, as described by you, connected with (what I at first supposed to be part of the uterus) the placenta.

"The child was born alive, appeared healthy, and lived thirty-six hours; but the placenta became extremely putrid before the child expired, which prevented my sending the whole in a better state of preservation.

"The mother is a thin, delicate woman, about twenty-two years of age; has been generally in good health, except having a cutaneous eruption, of which she has been cured near two years.

"She is in a situation of life not exposed to bodily fatigue or exertion, and not known to have entertained any improper mental presentiments during pregnancy. She cannot recollect having been surprised or frightened more than once during this period, and that was in the fifth month, when riding in a gig, the horse started and ran away, but she was neither thrown out nor hurt.

"Is it probable that the preternatural appearances in this case arose from the above fright?

"Is it your opinion that no malformation of the fœtus, or its extremities, could take place at that period of gestation?

"The quickness of the labour, (which was completed in less than an hour) I have no doubt arose from the singular adhesion of the head to the placenta, being the strongest possible stimulus to uterine action, during labour. Happy for me and my patient, that no part of the head presented. I observe, you have not noticed that the two first phalanges of the middle finger of the right hand were deficient.*

"Since the above case, a partial presentation of the placenta has fallen to my lot, and terminated favourably; but the child suffered so much, (I suppose from the loss of blood sustained in the delivery) that it only survived, in a lingering state, six weeks.

"The mother, who is perfectly recovered, was very near being the first midwifery patient I ever lost, during a tolerably extensive practice for the last ten years. And although I have conducted upwards of eighteen hundred labours, I only recollect four fœtal malformations, viz. a spina bifida; two monsters sent you; and the two fore-fingers of the right hand completely united.

"You

* Occasioned by my attention being engrossed in describing the head and upper parts of the body. T. P.

"You may communicate this letter to the public, if you think proper. I remain,

Your obliged friend,

CHARLES COOKE."

*Gloucester,*
*May* 3, 1800.

In anfwer to the queries propofed in the foregoing letter, viz. 1ft. "Is it probable that the preternatural appearances in this cafe arofe from the above fright?"

I can only anfwer briefly, that it does not appear to me probable that the appearances arofe from the caufe mentioned.

2d. "Is it your opinion that no malformation of the fœtus, or its extremities, could take place at that period of geftation?"

It is my opinion that no malformation of the fœtus, in any of its parts, can take place after it has once acquired its proper form, except what may arife from preffure, owing to fome unfavourable pofition in utero, or from inflammation of two furfaces in contact with each other.—The firft may produce incurvation, or other diftortion of parts.—The fecond, adhefions. Inftances of this latter we not unfrequently meet with; and it feems to have conftituted one of the peculiarities in the cafe before us, to wit, the adhefion of the head to the placenta.

To account for the various malformations which are produced in an endlefs variety, appears to me to be beyond the ftretch of finite wifdom.

I cannot fuppofe it poffible for the human mind, under any, even the moft violent impreffions, to disfigure the fœtus. If we could admit, as well authenticated indubitable facts, all the fanciful hiftories related by grave writers, refpecting the wonderful malformations in the human fœtus, as correfponding to previous mental impreffions, either from frights or longings, there would be no difficulty in admitting the mother's imagination to have a controul in the original formation, or in deforming it when once well formed.

None of my own patients, whom I have delivered of monftrous fœtufes, or other women with whom I have been acquainted, ever expreffed any apprehenfions of peculiar appearances in their children before delivery; but after they have been informed of the circumftances, they are prompt enough to recur to fome paft occurrence, in order to explain them, which had no affinity to the appearances in fuch children. On the other hand, a number of my own patients, as well as many others, have, from fome fhocks, or peculiar impreffions excited in their minds, had ftrong prepoffeffions that their children would be deformed or marked with correfponding impreffions; but in no one inftance have I ever known it to be the cafe.

We

We cannot possibly entertain the most distant idea, that when a child is produced with two heads, or without a head, that it has been occasioned by the mother's having longed for such a thing, or that she had seen such a child in the streets or elsewhere. Neither can a scientific man, possessed of his rational faculties, conceive it possibly in the power of the mother to add even a supernumerary finger or toe to a fœtus, in any stage of gestation, or to remove from the fœtus, and from the uterus, an extremity already well formed, or to transpose any of its parts; all of which every now and then occur to our notice. If we admit the mother's mind, imagination, or will, to have the power of performing such miraculous feats, with such an admirable dexterity, in the human species, we must go still further, and admit the same powers in the inferior parts of the creation; for we are presented with precisely similar deviations in quadrupeds, birds, &c. Some men, strenuous to establish their opinions in favour of the influence of the mother's mind, contend even for the possibility of these effects in such animals being produced by the same cause; and say, there is not that vast difference between reason and instinct, or between human ideas and those of other animals; that we arrogate too much, when we compare the perceptions of the human mind with those of some of the more sagacious quadrupeds. But we do not observe it to be among such that the extraordinary effects in question are produced. However, if they will contend that they arise from the causes assigned; if they will refer the multitude of phenomena to the powers of the mind, perhaps they will have no objection to carrying their favourite opinions one step further, into the vegetable kingdom, and suppose the innumerable instances of monstrosity, which daily occur to our notice, are produced there by the same cause, especially as there appears so great a similarity in these to those of animals, or, at least, as much so as the nature of the two can possibly admit. So that, after all that has been said by writers on both sides of the question, or perhaps all that can be offered on the subject, we must be content to sit down and confess, that the true cause is involved in inscrutable mystery, as are many more of Nature's laws. We can only view them as *lusus naturæ*; the true causes of which, or the manner in which the admirable powers of Nature are combined to effect them, will probably remain in the repository of her secrets to the end of time, as humiliating proofs of our limited comprehension.

One remark I have made of late is, that far the larger proportion of monsters are females, at least in the human subject. Those which I have collected, do not admit of a single exception; though I have certainly seen several instances of male monsters:

monſters: but thoſe deviations from the common mode of Nature's operations have been generally in the organs of generation, yet, in this reſpect too, the majority has been in females.

Theſe obſervations are not particularly addreſſed to my friend C. C. whoſe queries only demanded an affirmative or negative anſwer; but I have availed myſelf of this opportunity of contributing my part to the doing away of thoſe opinions which do not appear to be well founded, and which frequently excite conſiderable diſtreſs in the minds of pregnant women, who meet with alarming occurrences liable to agitate the mind, and thence give them painful apprehenſions of their being productive of ſome diſguſting formation in their children.

*Leadenhall Street, May* 10, 1800.

